# Microbial Technologies Employed for Biodegradation of Neonicotinoids in the Agroecosystem

**DOI:** 10.3389/fmicb.2021.759439

**Published:** 2021-12-02

**Authors:** Sajjad Ahmad, Dongming Cui, Guohua Zhong, Jie Liu

**Affiliations:** Key Laboratory of Integrated Pest Management of Crop in South China, Ministry of Agriculture and Rural Affairs, Key Laboratory of Natural Pesticide and Chemical Biology, Ministry of Education, South China Agricultural University, Guangzhou, China

**Keywords:** neonicotinoids, microbial degradation, metabolites, immobilization, non-target organisms

## Abstract

Neonicotinoids are synthetic pesticides widely used for the control of various pests in agriculture throughout the world. They mainly attack the nicotinic acetylcholine receptors, generate nervous stimulation, receptor clot, paralysis and finally cause death. They are low volatile, highly soluble and have a long half-life in soil and water. Due to their extensive use, the environmental residues have immensely increased in the last two decades and caused many hazardous effects on non-target organisms, including humans. Hence, for the protection of the environment and diversity of living organism’s the degradation of neonicotinoids has received widespread attention. Compared to the other methods, biological methods are considered cost-effective, eco-friendly and most efficient. In particular, the use of microbial species makes the degradation of xenobiotics more accessible fast and active due to their smaller size. Since this degradation also converts xenobiotics into less toxic substances, the various metabolic pathways for the microbial degradation of neonicotinoids have been systematically discussed. Additionally, different enzymes, genes, plasmids and proteins are also investigated here. At last, this review highlights the implementation of innovative tools, databases, multi-omics strategies and immobilization techniques of microbial cells to detect and degrade neonicotinoids in the environment.

## Introduction

Since the global implementation of synthetic insecticides-centered strategies for pest prevention from the 1960s, the satisfaction of increase in crop yield has been compromised by the unexpected pest resistance against mainstream insecticides such as organophosphates (OPs), carbamates, and pyrethroids (Jayaraj et al., 2016). As a promising alternative, neonicotinoid pesticides (Table 1) that mainly target the nicotinic acetylcholine receptor (nAChR) and impact nervous, sympathetic, and parasympathetic systems of insects were first launched in the 1990s (Sparks and Nauen, 2015). Chemical, physical and biological properties of neonicotinoids were collected from Pesticide Properties Database (PPDB^1^). Currently, this group has been developed as the most intensively used insecticide globally, authorized for more than 140 crops in about 120 countries (Table 2; Wood and Goulson, 2017; Zhang et al., 2018a).

**TABLE 1:** Neonicotinoid compounds their chemical structures, physical state, molecular mass, molecular formula, melting point and water solubility.

Compound name	Physical state	Molecular formula	Molecular mass (g⋅mol^–1^)	Melting point (°C)	Water Solubility (mg/L)	Chemical structure
Acetamiprid	White crystals, white fine powder, odorless	C_10_H_11_ClN_4_	222.68	98.9	2950	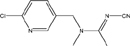

Clothianidin	Clear colorless solid powder, odorless	C_6_H_8_ClN_5_O_2_S	249.68	176.8	304	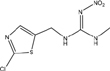

Cycloxaprid	Wettable powder	C_14_H_15_ClN_4_O_3_	322.75	-	-	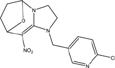

Dinotefuran	White crystalline solid, odorless	C_7_H_14_N_4_O_3_	202.21	107.5	39830	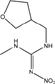

Imidacloprid	Clear crystals or beige powder	C_9_H_10_ClN_5_O_2_	255.67	144	610	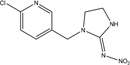

Nitenpyram	Pale yellow crystals	C_11_H_15_ClN_4_O_2_	270.72	82	590000	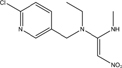

Sulfoxaflor	White solid	C_10_H_10_F_3_N_3_OS	277.27	112.9	568	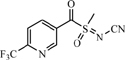

Thiacloprid	Yellow crystalline powder, odorless	C_10_H_9_ClN_4_S	252.72	136	185	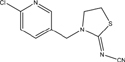

Thiamethoxam	Slightly creamy crystalline powder, odorless	C_8_H_10_ClN_5_O_3_S	291.71	139.1	4100	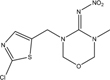

*Source: Casida, 2011; Sheets et al., 2016; Pang et al., 2020; Pietrzak et al., 2020; and Data from Hazardous Substance Data Bank (HSDB), available at: https://sitem.herts.ac.uk/aeru/ppdb/en/index.htm (Accessed: 23 May 2021).*

**TABLE 2:** Typical neonicotinoid insecticides and their basic characteristics.

Category	Name	Year of registration	Target arthropods	Crops	References
First generation (chloropyridinyl compounds)	Imidacloprid Nitenpyram Acetamiprid Thiacloprid	1992 1995 2002 2003	Whiteflies, termites, beetles, fleas, ants, bugs, centipedes, cockroaches, crickets, earwigs, flies, millipedes, mosquitoes, moths, scorpions, silverfish, spider mites, spiders, ticks, wasps and aphids	Ornamental plants, cotton, rice, cereals, peanuts, vegetables, pome fruits, pecans,	Shao et al., 2008; Fairbrother et al., 2014; Hussain et al., 2016; Thompson et al., 2020

Second generation (chlorothiazolyl compounds)	Thiamethoxam Clothianidin	2001 2003	Aphids, thrips, beetles, centipedes, millipedes, sawflies, leaf miners, stem borers, termites. flies, moths and true bugs	Vegetables, ornamental plants, citrus, cotton, rice, corn, tobacco, canola, grapes	Fairbrother et al., 2014; Zhao et al., 2020

Third generation (tetrahydrofuryl compound)	Cycloxaprid Dinotefuran Sulfoxaflor	2008 2012 2013	Aphids, whiteflies, thrips, leafhopper, planthoppers, leaf miner, sawfly, mole cricket, white grubs, bugs, psyllids, beetles, mealybugs, sawfly larvae and cockroaches.	Cotton, rice, mustard, turf, lawn & garden, vegetable crops, alfalfa, cacao, citrus, corn, cucurbits, grains, pineapples	Hussain et al., 2016; Liao et al., 2019

**FIGURE 1:**
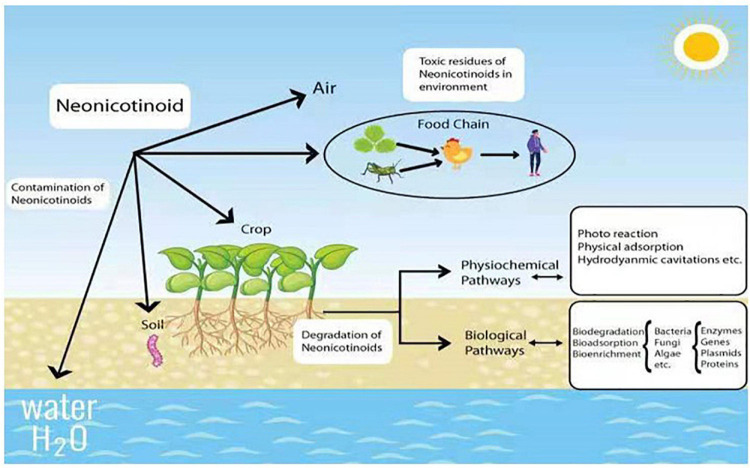
Neonicotinoid contamination and their degradation by various methods.

In general, neonicotinoid insecticides are mainly used for sucking, boring, and root-feeding pests like Lepidoptera, Coleoptera, and Hemiptera, respectively (Cimino et al., 2017; Halsch et al., 2020). Since some Hemipteran insects (like aphids) can spread viruses in plants, this group is also recommended against the spread of disease viruses in crops (Simon-Delso et al., 2015). Additionally, neonicotinoids are used for wood protection against the attack of termites (Hano et al., 2019). Due to broad-spectrum and mode of action, they are highly employed as veterinary medicines to prevent different animal pests like fleas, ticks, and worms (Pisa et al., 2015). However, only 5% of neonicotinoid pesticides can reach specific targets during field application (Acero et al., 2019). Due to the extensive consumption, the residues of neonicotinoids are ubiquitously found in the environment at the levels of parts per billion and million (ppb-ppm) in soil and plants, while it is parts per trillion (ppt) in water (Bonmatin et al., 2015; Morrissey et al., 2015), which become a serious threat to a wide range of non-targeted organisms (Figure 1).

The typical neonicotinoids, such as imidacloprid and clothianidin, produce sub-lethal effects on other living organisms, including genotoxicity, cytotoxicity, immunosuppression, reduced growth, and reproduction disorder in the vertebrates (Gibbons et al., 2015; Han et al., 2018). Recently, Schulz et al. (2021) found that the neonicotinoid exposure and toxicity have increased significantly to the aquatic invertebrates, pollinators, genetically modified crops (corn and soybeans), and terrestrial plants. It has been reported that neonicotinoid residues at high levels may seriously threaten the vertebrates’ reproductive and developmental systems (fish, mammals, birds, reptiles, amphibians) and even humans (Berheim et al., 2019). Neonicotinoids are also evaluated to increase the aromatase expression to participate *in vitro* models related to human breast cancer development and change the function of critical antioxidant enzymes like catalase, superoxide, dismutase, and glutathione peroxidase (Annabi et al., 2015; Caron-Beaudoin et al., 2018). More alarmingly, increasing shreds of evidence showed that the intensive use of neonicotinoids at the flowering and blooming stages might become the major cause for the colony collapse disorder within the predators and pollinators, especially bees (Woodcock et al., 2017). Mitchell et al. (2017) found that due to the imprudent use of neonicotinoids (acetamiprid, clothianidin, imidacloprid thiacloprid, and thiamethoxam), the habitat of pollinators and the quality of honey were severely affected. Around the globe, 198 honey samples were analyzed, among 75% contained at least one class of neonicotinoid, while 45% contained two or five and 10% contained four or five in quantifiable amounts. Moreover, this study indicated that the North American (86%), Asian (80%), and European (79%) regions had the ubiquitous existence of neonicotinoid residues in honey samples.

To alleviate the environmental toxicity of neonicotinoids, effective, low-cost, and sustainable methods are urgently needed to degrade neonicotinoid residues. In general, the reduction of toxic substances from the environment can be achieved by several methods such as physical adsorption and advanced oxidant process (Ye et al., 2019). Among all the techniques, biodegradation by microorganisms is considered to be more efficient, reliable, eco-friendly, and cost-effective (Li et al., 2020). For decades, a large variety of microbial species have been discovered for the degradation of pesticide residues. More importantly, many isolates reveal attractive properties such as the complete mineralization of pesticides and their toxic metabolites, endowing the approach of microbial degradation with high potential for future practical application (Yue et al., 2018).

To achieve the effective degradation of neonicotinoids, the study of enzymes involved in its metabolism imparts foundational insights for understanding the transformation pathway, which ultimately influences the efficacy of the biodegradation strategy. In particular, a cell-free extract containing abundant intracellular enzymes has been widely employed as one of the most versatile and efficient techniques. Furthermore, recent molecular advances have empowered omics approaches and databases to provide new insights that contribute to the discipline of biodegradation by allowing concurrent analysis of millions of microbes and cover the path of pesticide biodegradation. Hence, this review aims to summarize the strength and molecular basis of microbial degradation for neonicotinoid pollution. Also, we focus on the recent development of microbial active element-centered techniques using whole cells, enzymes, genes, plasmids, and modern biological molecular tools, which establish a myriad of promising strategies to promote the potential for environmental monitoring and biodegradation neonicotinoids.

## Potential Microorganisms for Neonicotinoid Degradation and Their Metabolic Pathways

Functional microbes, mainly bacteria and fungi isolated from contaminated soil, water, and sediment, are considered one of the most versatile candidates for the efficient and effective biodegradation of persistent pesticides in the agricultural ecosystem (Kumar et al., 2019). The extensive use of neonicotinoids in various crops has raised a significant concern due to their toxicity and persistence in the ecological system and human health. Recently, Menon et al. (2021) proposed a study about the perseverance of different types of neonicotinoids in the water soil systems of the paddy fields in the Cauvery delta region, South India. This study revealed that the neonicotinoids are less persistent in the water soil systems, and they are readily exposed to photolysis and undergo efficient microbial degradation. Moreover, hydropedological characteristics of highly saturated delta soil enable their leaching into groundwater by vertical migration and infiltration.

Various studies have confirmed that a wide range of microbes are capable of degrading neonicotinoids in not only liquid cultures but also in a contaminated environment (Table 3). Recently, García-Galán et al. (2020) degraded various types of neonicotinoids (acetamiprid, clothianidin, imidacloprid, thiacloprid, and thiamethoxam) through microalgae species (*Ulothrix sp.*, *Oocystis sp.*, and *Synechocystis sp.*) in wastewater using semi-closed, tubular horizontal photobioreactor (PBR). This study highlighted new data on the ability of microalgae-based treatment systems to degrade not only different types of neonicotinoids but also non-targeted pesticide transformation products or their intermediates into the original compounds using PBR. Furthermore, this study could provide a practical and feasible explanation even in higher concentrations in the effluents under real environmental conditions.

**TABLE 3:** The ability of isolated microorganisms in degrading various neonicotinoids.

Target neonicotinoid	Microorganisms	Source	Degradation (%)	References
Acetamiprid	*Pigmentiphaga sp.* strain D-2	Grow in laboratory	99	Yang H. et al., 2020
	*Streptomyces canus*	Soil	90.32	Guo et al., 2019
	*Ensifer adhaerens*	Soil	87.8	Sun et al., 2018
	*Fusarium sp.*	Contaminated soil	99.6	Shi et al., 2018
	*Staphylococcus aureus strain 502*	Wetland wastewater	61.68	Kanjilal et al., 2016
	*Pseudoxanthomonas sp.* AAP-7	Polluted soil	100	Wang et al., 2013b
	*Pigmentiphaga sp. D-2*	Wastewater	99	Yang et al., 2013
	*Rhodococcus sp.* BCH-2	Contaminated soil	84.65	Phugare and Jadhav, 2015
	*Stenotrophomonas sp.* THZ-XP	Sludge	100	Tang et al., 2012
	*Pseudomonas aeruginosa* BCRC 11864	Commercial	76.55	Toolabi et al., 2017
Clothianidin	*Ochrobactrum anthropi, Acinetobacter johnsonii, Pseudomonas sp.* and *Stenotrophomonas maltophilia*	Vegetable green house	79.3	Wang et al., 2018
	*Pseudomonas stutzeri*	Soil	62	Parte and Kharat, 2019
	*Rotaria, Suctorida*, and *Vorticella sp.*	Municipal wastewater	88	Racar et al., 2020
Dinotefuran	*Phanerochaete sordida* YK-624	Rotten wood	100	Wang et al., 2019b
	*Methylotenera sp., Ramlibacter sp., Rubrivivax sp.* and *Nitrospira sp.*	Soil	28.8-34.3	Yu et al., 2020
Imidacloprid	*Bacillus aerophilus*	Sandy loam soil	81.20-99.14	Akoijam and Singh, 2015
	*Methylobacterium radiotolerans* and *Microbacterium arthrosphaerae*	Corn field	88.4-98.7	Erguven and Yildirim, 2019
	*Pseudomonas, Enterobacter, Aspergillus and Rhodotorula*	Strawberry field	80	Li et al., 2020
	*Mycobacterium sp.*	Wheat and clover field	99.7	Kandil et al., 2015
	*Hymenobacter latericoloratus* CGMCC	Water	52.4-68.2	Guo et al., 2020
	*Aspergillus terreus* YESM3	Wastewater drains	96.23	Mohammed and Badawy, 2017
	*Ochrobactrum BCL-1*	Tea rhizosphere soil	78	Hu et al., 2013
	*Pseudomonas sp.* RPT 52	Agriculture field	46.5	Gupta et al., 2016
	*Rhizobium sp.*	Vegetable farming areas	45.48	Sabourmoghaddam et al., 2015
	*Pseudoxanthomonas indica* CGMCC 6648	Rhizospheric soils	70.1	Ma et al., 2014
	*Bacillus alkalinitrilicus*	Sugarcane field	93.8	Sharma et al., 2014a
	*Phanerochaete chrysosporium*	Rotten wood	97	Mori et al., 2021
	*Bacillus aerophilus* and *Bacillus alkalinitrilicus*	Soil	30-40	Sharma et al., 2014b
	*Bacillus thuringiensis*	Marine sediment	78	Ferreira et al., 2016
Nitenpyram	*Phanerochaete sordida* YK-624	Rotten wood	100	Wang et al., 2019b
	*Rhodococcus ruber* CGMCC 17550	Sewage	98.37	Dai et al., 2021
	*Aspergillus sp.*	Commercial formulation biotechnology	92.9	Chen et al., 2019
Sulfoxaflor	*Aminobacter sp.* CGMCC 1.17253	Agriculture soil	59.1	Yang W. L. et al., 2020
Thiamethoxam	*Acinetobacter sp., Enterobacter sp.* and *Bacillus sp.*	Soil	82.06-94.72	Hegde et al., 2017
	*Enterobacter sp*. TMX13	Roots of mulberry (*Morus alba* L.)	85.2	Wang W. et al., 2020
	*Bacillus aeromonas* IMBL 4.1, *Pseudomonas putida* IMBL 5.2, *Acinetobacter sp.* TW and *Sphingomonas sp.* TY	Agriculture soil	38.23-45.28	Rana et al., 2015
	*Ensifer adhaerens* TMX-23	Agriculture soil	81	Zhou et al., 2013
	*Enterobacter cloacae* TMX-6	Rice field	99	Zhan et al., 2021
	*Pseudomonas fluorescens*	Isle cultures	67	Zamule et al., 2021
	*Pseudomonas putida*	Isle cultures	65	Zamule et al., 2021
	*Escherichia coli*	Isle cultures	60	Zamule et al., 2021
	*Pseudomonas sp.* 1G	Soil	70	Pandey et al., 2009
	*Phanerochaete chrysosporium*	Commercial	98	Chen et al., 2021
	(Mix microbial culture of genera) *Achromobacter, Agromyces, Ensifer, Mesorhizobium, Microbacterium* and *Pseudoxanthomonas*	Soil	96	Zhou G. C. et al., 2014
Thiacloprid	*Ensifer meliloti* CGMCC7333	Rhizosphere soils	86.8	Ge et al., 2014
	*Stenotrophomonas maltophilia CGMCC*1.178	China general microbiological (CC)	100	Zhao et al., 2009
	*Variovorax boronicumulans* J1	Agricultural soils	62.5-100	Zhang et al., 2012
	*Microvirga flocculans* CGMCC 1.16731	Contaminated soil	92.4	Zhao et al., 2019b
	*Phanerochaete chrysosporium*	Rotten wood	74	Mori et al., 2021

Biodegradation of neonicotinoids and other pesticides in the groundwater occurred in the absence or deficiency of oxygen conditions with the correlation of anaerobic microorganisms (Pietrzak et al., 2020). The biodegradation of clothianidin under anaerobic and aerobic conditions was studied by Mulligan et al. (2016). Various parameters were also investigated, such as the effect of nutrients, concentration of pesticide, and temperature. Results showed that the clothianidin degradation rate was higher in anaerobic conditions than aerobic conditions at different temperature ranges. Monsalvo et al. (2014) designed an experiment for the anaerobic biodegradation of imidacloprid in an expanded granular sludge bioreactor (EGSB) and revealed that imidacloprid dramatically decreased in EGSB. After 30 days, for the degradation of imidacloprid with the concentration of 20 mg/L, the EGSB gained a stable chemical oxygen demand of 0.9 g CH_4_-COD/g COD and methane production of around 85%, respectively. In another study, Gupta et al. (2008) found anaerobic conditions for microbial species to degrade thiamethoxam, resulting in DT_50_ values of 46–75 days that are more effective, fast, and efficient. For the resourceful removal of neonicotinoids in agriculture wastewater, Rodríguez-Castillo et al. (2019) isolated eight bacterial and one yeast strain. The biological process was sealed up in batch stirred tank bioreactors and degraded mixtures of neonicotinoids (imidacloprid + thiamethoxam and imidacloprid + thiamethoxam + acetamiprid) 95.8% and 94.4% of total neonicotinoids, respectively.

Microbes and duckweed species (*Lemna turionifera* and *Ceratophyllum demersum*) for the biotransformation of imidacloprid and thiacloprid were collected from pond water. The results of this study explained that the removal of tested neonicotinoids required the presence of duckweed and its associated microbial community, which suggested that this mechanism was synergistic. In the presence of duckweed and microbes, imidacloprid and thiacloprid were efficiently degraded in the hydroponic medium at the rates 0.63 ± 0.07 and 0.62 ± 0.05 per day, respectively. Furthermore, their degradation (imidacloprid and thiacloprid) converted into multiple intermediates such as desnitro-imidacloprid, imidacloprid urea, thiacloprid amide, and 6-chloronicotinic acid. This novel study provides new insights to remove hazardous substances and excellent contribution to the environmental fate of neonicotinoids (Muerdter and LeFevre, 2019). Another study removed neonicotinoids (acetamiprid, imidacloprid, thiamethoxam) and other organic and inorganic emerging pollutants by a monoculture of *Chlorella vulgaris* and mixed microalgal-bacterial culture for the conventional wastewater treatment. Both *C. vulgaris* and mixed microalgal-bacterial batch cultures could remove neonicotinoids and other hazardous pollutants. The monoculture of microalgal degrades (imidacloprid and acetamiprid) rapidly and proposed various transformation pathways, suggesting that increasing algae concentration in full-scale algae-based treatment systems can improve their removal rate and efficiency. The results of this study also suggested that environmentally relevant spiked concentrations (1–20 μg/L) did not inhibit the growth of micro-algal and *C. vulgaris* cultures (Prosenc et al., 2021). A yeast was identified instead of bacterial and fungal strains for the degradation of different classes of neonicotinoids such as acetamiprid, imidacloprid, imidaclothiz and thiacloprid as *Rhodotorula mucilaginosa* strain IM-2. Results showed that yeast strain efficiently degrade acetamiprid and thiacloprid in sucrose mineral salt medium with half-lives of 3.7 and 14.8 days, respectively. At the same time, no degradation was observed in the case of imidacloprid and imidaclothiz (Dai et al., 2010).

Guo et al. (2020) evaluated oligotrophic bacterial strain *Hymenobacter latericoloratus* CGMCC 16346 to degrade imidacloprid by co-metabolism hydroxylation. The bacterial cells degraded 64.4% of 100 mg/L imidacloprid in surface water. After the incubation of 6 days in the existence of maltose as co-substrate, bacterial enriched culture degraded 40.8% of imidacloprid in 10 days. The bacterium removed imidacloprid in surface water without co-substrate supplementation and retained imidacloprid-degrading activity after 30 days. Recently, in another study, Encarnação et al. (2021) isolated a microalgae *Nannochloropsis sp.* from wastewater and investigated it for the bioremediation of imidacloprid with the initial concentration of 9.59 μg/mL. Results demonstrated that within 20 h, microalgae degraded 50% of imidacloprid in synthetic wastewater. Chiral insecticide paichongding is a kind of neonicotinoid promising to replace imidacloprid. Its prominent features include being less toxic to mammals and broad-spectrum mode of action against sucking and biting insect pests widely used in China (Lou et al., 2015). Due to the dramatic effects on the environment, soil microbial community and soil enzyme activity are severely affected by the parent compound and its by-products (Cai et al., 2016; Bian et al., 2018). Wang J. et al. (2016) studied its remediation in an aqueous environment using two bacterial species (*Sphingobacterium sp.* G1-13 and G1-14), which degrade 35% in 5 days. Furthermore, in this study, metabolites were also detected and two possible degradation pathways are proposed, which provide new insights into the biodegradation of novel types of neonicotinoids.

To degrade imidacloprid residues using microbial species and to investigate the environmental parameters such as chemical oxygen demand (COD) and biochemical oxygen demand (BOD_5_), a study was carried out by Erguven and Yildirim (2019). Two bacterial species (*Methylobacterium radiotolerans* and *Microbacterium arthrosphaerae*) were isolated from the corn field in the Thrace region of Turkey, and both were cultured on plate count agar at 28°C. After growth of 7 days, 10^7^ CFU/ml suspensions of each bacterium were used to prepare 100 ml sterile flasks containing 99 ml sabouraud dextrose broth. Further, the mass of flasks was homogenized and designed three different concentrations, 20 ml, 40 ml, and 80 ml, to remove imidacloprid at the concentration of 700 ppm. For the biodegradation experiment, three flasks were treated with sterile corn farming soil, mixed bacterial culture, and imidacloprid. In comparison, the blank bottle was treated with imidacloprid and sterile soil. All the flasks were diluted using a 250 ml distilled water. After 18 days, the water filtered sample result showed that mixed microbial culture was able to degrade 98.7%, 96.4%, and 51.6% of imidacloprid, respectively. In the case of BOD_5_, the same concentrations of mixed microbial culture were able to remove 88.4%, 78.6%, and 49.9% of imidacloprid, respectively. This study concluded that mixed cultures of bacterial species with a concentration of 80 ml and 40 ml provide high degradation rate than the low concentration (20 ml).

To degrade imidacloprid and acetamiprid residues in the environment, a bacterial strain *Stenotrophomonas maltophilia* CGMCC 1.1788 was used and found that this strain was efficiently involved in the degradation process and detoxified both compounds. The difference in metabolism and detoxification pathways of both pesticides appeared due to structural differences (Chen et al., 2008). In another study same bacterial strain was used by Dai et al. (2006) for the removal of imidacloprid and found that resting cells of this bacterial strain convert imidacloprid into 5-hydroxyl IMI [1-(6-chloro-3-pyridylmethyl)-5-hydroxyl-N-nitroimidazolidin-2-ylideneamine] at the maximum conversion rate.

Guo et al. (2019) monitored the degradation of another neonicotinoid, acetamiprid, in the environment *via* actinomycete *Streptomyces canus* CGMCC 13662 enriched from the agricultural soil. After 48 h of incubation, at 200 mg/L, about 70% degradation of acetamiprid was achieved. The degradation of thiamethoxam was investigated in municipal wastewater, and the microbial action accelerated dissipation by UV light to degrade acetamiprid in the contaminated environment, a bacterial strain ACE-3 was isolated from soil, and results indicated that this strain was able to degrade acetamiprid entirely at the initial concentration of 50 mg/L within 144 hours in a broad range of environmental conditions such as pH (6–8) and temperature (20–42 °C) (Peña et al., 2011; Xu et al., 2020).

For the recovery of clothianidin and acetamiprid, Harry-Asobara and Kamei (2019) isolated the bacterial species (*Enterobacter sp.* TN3W-14 and *Pseudomonas sp.* TN3W-8) and white-rot fungal specie (*Phlebia brevispora*) strains TN3F and TMIC33929. The bacterial strains used are promoting the growth of fungal hyphae, and it was shown that growing co-cultures of *Phlebia* and these bacteria appeared to improve the degradation of neonicotinoids based on the decreased recovery of the compounds compared to either the axenic fungal culture or the bacterial cultures.

### The Microbial Metabolic Pathways for Neonicotinoid Biodegradation

The excessive use of neonicotinoids has generated long-term toxic residual contamination in the environment (Shahid et al., 2021). More importantly, when various microbes convert the parent compounds, it may lead to intermediates with the same or higher toxicity than the parent compounds (Li, 2021). Consequently, a comprehensive understanding of neonicotinoid biodegradation pathways would help to better utilize these functional microbes for more sustainable remediation.

**FIGURE 2:**
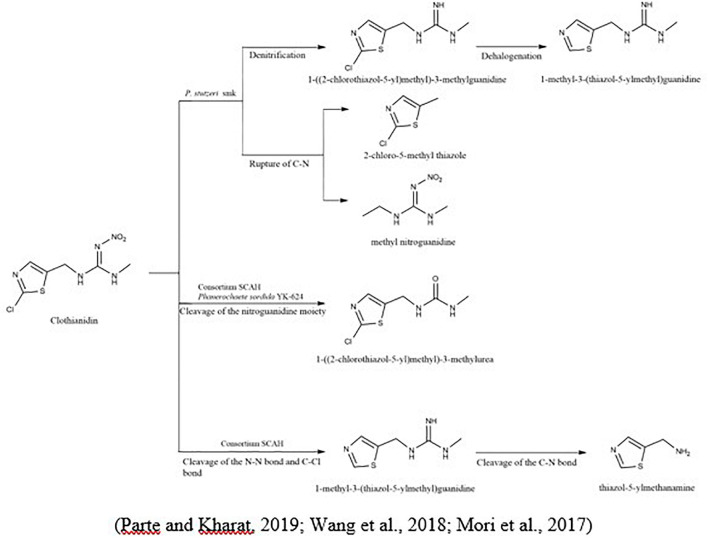
Microbial metabolic pathway of clothianidin and their intermediate products.

To degrade clothianidin, the breakdown of C-N bonds between thiazolyl methyl and the guanidine division delivers carbon for the transformation of clothianidin to 2 chloro-5-methyl thiazole and methyl nitroguanidine (Parte and Kharat, 2019). The microbial method slowly degraded clothianidin to ((2-chlorothiazol-5-yl) methyl-3-methylguanidine and methyl-3-(thiazol-5-yl) methyl) guanidine *via* denitrification and dehalogenation (Pang et al., 2020). Mori et al. (2017) studied the clothianidin’s degradation and metabolic pathways using pure culture white-rot fungus, *Phanerochaete sordida*, inoculated from the rotten wood. The degradation metabolite of clothianidin N-(2-chlorothiazol-5-yl-methyl)-N′-methyl urea was determined using high-resolution electrospray ionization mass spectrometry technique. Wang et al. (2019c) also reported the degradation of clothianidin. Still, the difference from the above study is the use of bacteria instead of fungi, and results revealed that this compound degraded into further three metabolites, while the one metabolite is identical in the study as mentioned above. The other two metabolites are N-(1, 3-thiazole-5-ylmethyl)- N′-methyl guanidine and 5-amino-methlthiazol. The bacterial strains used in this study (*Ochrobactrum anthropi*, *Acinetobacter johnsonii, Pseudomonas sp*., and *Stenotrophomonas maltophilia*) provided the highest degradation of 79.3% in the mineral salt medium after the incubation of 15 days. So, this could be due to the use of different microbial species, which give the various metabolites of the same compound (Figure 2).

**FIGURE 3:**
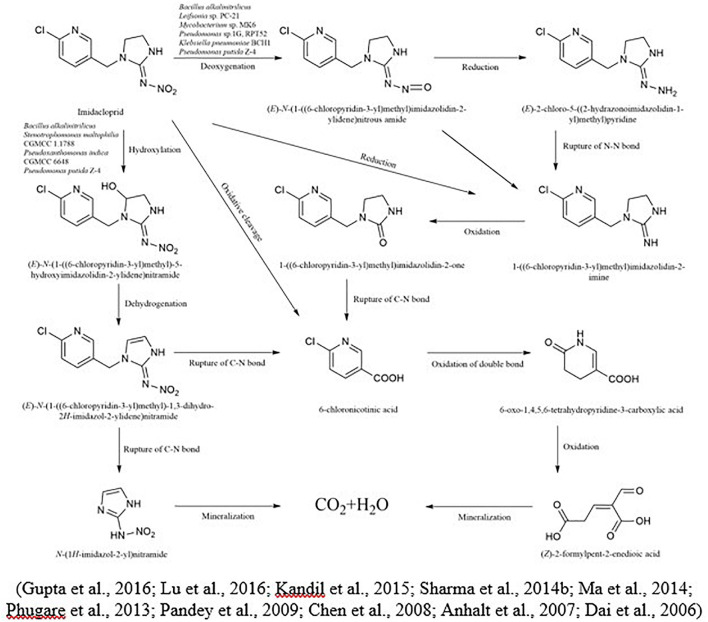
Microbial metabolic pathway of imidacloprid and their intermediate products.

Imidacloprid is highly persistent in soil with a half-life of more than 100 days (Anhalt et al., 2007). In the case of imidacloprid, oxidation and nitro reduction are the two most essential pathways (Lu et al., 2016). Imidacloprid cleavages into metabolites like urea, 6-chloronicotinic aldehyde, 6-chloro-N-methylnicotinacidamide, and 6-chloronicotinic acid (Sabourmoghaddam et al., 2015; Fusetto et al., 2017). Three main different biological degradation pathways can degrade the imidacloprid: hydroxylation of the imidazolidine ring, reduction of the nitro group, and loss of the nitro group (Figure 3). In another study for the degradation of imidacloprid, a bacterial strain *Klebsiella pneumoniae* strain BCH1 was isolated. After optimizing bacterial strain on various environmental conditions, results showed that it was more active at 30 °C and degraded approximately 78% of imidacloprid within a week. Moreover, in this study using gas chromatography and mass spectrometry, three metabolites were identified as nitrosoguanidine, imidacloprid guanidine and 6-chloronicotinic acid and toxicity of parent compound and their intermediates were tested using model insect silkworm (*Bombyx mori*) (Phugare et al., 2013). Ma et al. (2014) proposed the metabolic pathway of imidacloprid by using consortium *Pseudoxanthomonas indica* CGMCC 6648. In the existence of glucose, the imidacloprid can be degraded to 5-hydroxy imidacloprid and imidacloprid olefin can be degraded in 6 days. In the presence of lactose, imidacloprid can be degraded to 5-hydroxy imidacloprid in 48 h. However, in the presence of pyruvate, it can form olefin imidacloprid in 96 h. *P. indica CGMCC* 6648 simultaneously degraded the imidacloprid and formed olefin imidacloprid. In another study, Sharma et al. (2014a) also studied the degradation of imidacloprid by *Bacillus alkalinitrilicus* and proposed possible intermediates such as 6-chloronicotinic acid, nitrosimine, and imidacloprid-NTG.

**FIGURE 4:**
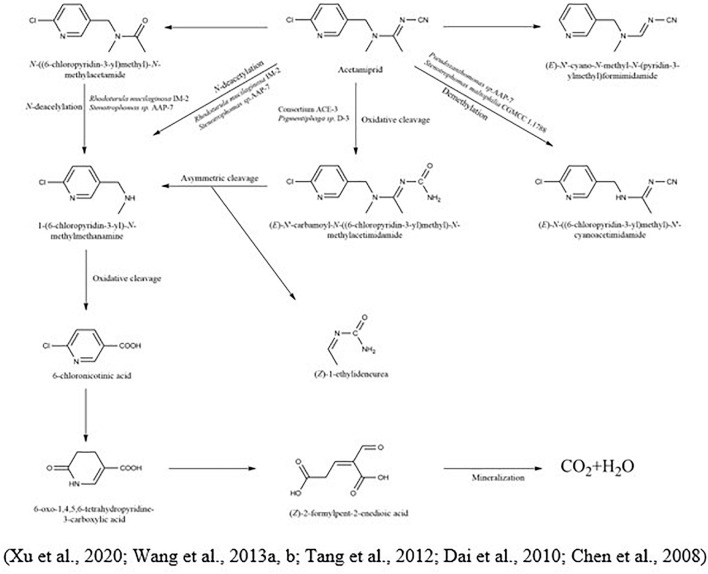
Microbial metabolic pathway of acetamiprid and their intermediate products.

The degradation of acetamiprid and its metabolic pathway was evaluated by Sun et al. (2018) using *Ensifer adhaerens* CGMCC 6315, which rapidly degraded 87.8% of acetamiprid-polluted soil (at the initial concentration of 5 mg/kg) within 2 days. During the microbial degradation of acetamiprid, the triple bond between carbon and nitrogen of the compound was oxidized and splintered to produce N-amidoamine derivatives. Due to the uneven breakdown, the product was divided into N-methyl-(6-chloro-3-pyridyl) methylamine and (Z)-1-ethylideneurea. The metabolites rapidly produced 6-chloronicotinic acid, which was finally mineralized to H_2_O and CO_2_. In another study, Shi et al. (2018) demonstrated the metabolic pathway of acetamiprid *via Fusarium sp.* strain. CS-3 N′-[(6-chloropyridin-3-yl) methyl]-N-methylacetamide, 2-chloro-5-hydroxymethylpyridine, and 6-chloronicotinic acid were distinguished as the most prevailing intermediates (Figure 4).

**FIGURE 5:**
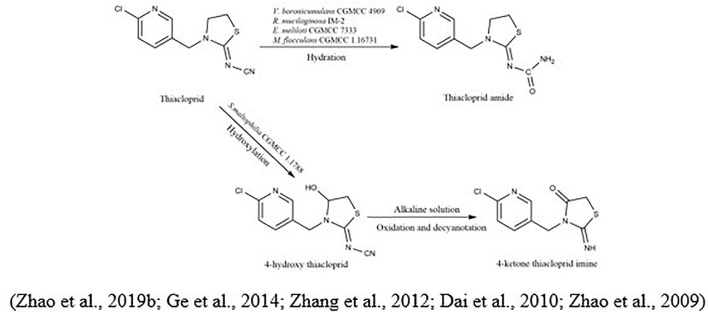
Microbial metabolic pathway of thiacloprid and their intermediate products.

The metabolic pathway of thiacloprid was also reported. Zhang et al. (2018b) showed that at least eight bacterial genera were linked to the degradation of thiacloprid into its intermediate thiacloprid amide, which would be related to oxidative cleavage. Recently, Zhao et al. (2019b) demonstrated the transformation of thiacloprid *via* nitrogen-fixing bacterium *Microvirga flocculans* CGMCC 1.16731 through the process of hydroxylation and hydration to thiacloprid amide and 4-hydroxy thiacloprid. A cobalt-type nitrile hydratase (nhase) is formed by two subunits, such as α-subunit (*tnhA*) and a β-subunit (*tnhB*). Both subunits transformed thiacloprid to thiacloprid amide (Figure 5).

**FIGURE 6:**
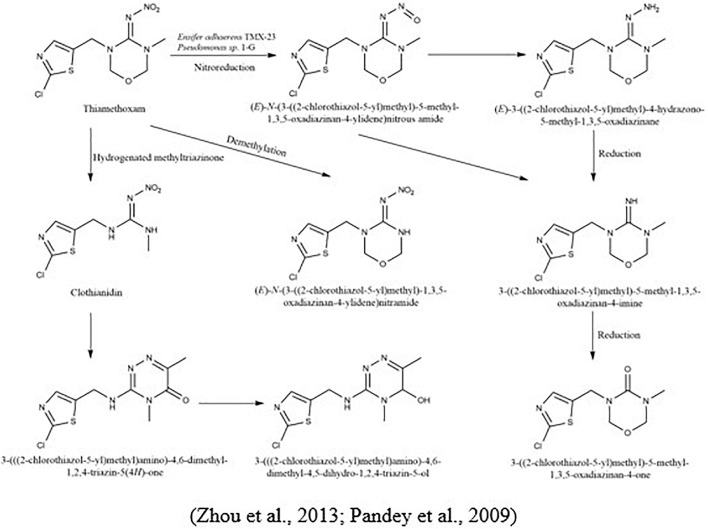
Microbial metabolic pathway of thiamethoxam and their intermediate products.

Thiamethoxam, 3-(2-chloro-1,3-thiazol-5-yl-methyl)-5-methyl-1,3,5-oxadiazinan-ylidene (nitro) amine, is another type of neonicotinoid pesticide that shares the same mechanisms as imidacloprid and has no interactive resistance to imidacloprid, idinidine, or alkenididine (Pang et al., 2020). Pandey et al. (2009) studied the microbial degradation of thiamethoxam and imidacloprid by *Pseudomonas sp.* 1G, and the results of the study revealed that both pesticides were transformed to nitrosoguanidine ( = N-NO), desnitro ( = NH), and urea ( = O) metabolites. In another study, a demethylation pathway was adopted to degrade thiamethoxam, forming desmethyl-thiamethoxam (Zhou et al., 2013; Figure 6).

**FIGURE 7:**
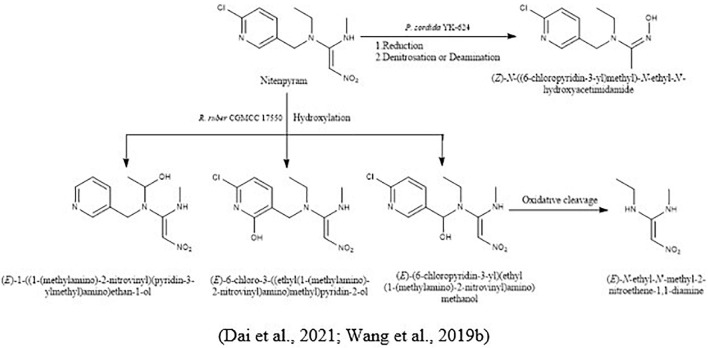
Microbial metabolic pathway of nitenpyram and their intermediate products.

For the biodegradation of nitenpyram and dinotefuran, Wang et al. (2019b) isolated a white-rot fungus, *Phanerochaete sordida* YK-624, and proposed their microbial metabolic pathways. Nitenpyram converted into their intermediate (Z)-N-((6-chloropyridin-3-yl) methyl)-N-ethyl-N′-hydroxyacetimidamide in two ways, reduction of nitenpyram and by denitrosation or deamination. In the opening ring of the hydroxyl group, by hydroxylation and dehydration pathway, dinotefuran was converted into its metabolites (Figure 7).

## Microbial Technologies for the Enhanced Neonicotinoid Biodegradation

There are many different conventional methods and combined technologies used to eliminate toxic pesticide residues from the agroecosystem. These biodegradation methods strive to clean up and transform a massive range of accumulated poisonous compounds in the environment (John et al., 2018). With the improvement of scientific methods by various researchers in biodegradation, gene editing and systematic biological tools are being used to eliminate not only pesticides but also other hazardous wastes (Malla et al., 2018). Using microbial technologies, many effective microbes and their related genes, plasmids, and enzymes useful in biodegradation processes with precise information are identified. The use of microbial technologies fixes the degradation of microbial species and their co-related parts, which are used to deal with pesticide polluted agroecosystem. In recent years, molecular, omics, and database approaches together with immobilization techniques are playing a vital role in the analysis of microbial communities in the contaminated site. These novel techniques provide incredible insights into key biodegradative pathways and the ability of microbes to accept environmental stress conditions. These approaches facilitate the explanation of microbial species at the taxonomic level and provide new insights to access the pool of genetic resources for the development of biodegradation in an agroecosystem (Czaplicki and Gunsch, 2016).

### Molecular Approaches

Nowadays, as compared to the common biological pathways, traditional and advanced molecular biological techniques such as clone libraries, probes, reverse sample genome probing, fluorescent *in situ* hybridization, community profiling or DNA fingerprinting, next-generation sequencing, pyrosequencing analysis, single-cell genome sequencing, and massively parallel signature sequencing provide a more significant explanation, identification of entire profile, and complete biodiversity of microbial communities (Bailón-Salas et al., 2017). Various techniques associated with enzymes, genes, and DNA of microbes such as genes (plasmids and transposons) are responsible for removing pesticides (Parween et al., 2016). The various molecular strategies which are used for the effective biodegradation of pesticides are restriction fragment length polymorphism (RFLP), dot blot, Southern blot, polymerase change reaction (PCR) amplification, subsequent analysis of bacterial rRNA genes by sequencing, preparing metagenomic libraries, denaturing gradient gel electrophoresis (DGGE), and microarrays (Sinha et al., 2009). Recently, Mori et al. (2021) had studied the degradation of various neonicotinoids by white-rot fungus, *Phanerochaete chrysosporium*, and identified cytochrome P450 enzyme and two major isoenzymes (CYP5037B3 and CYP5147A3) involved in the metabolism of three neonicotinoids (acetamiprid, imidacloprid, and thiacloprid). Both isoenzymes catalyzed the breakdown of the chloropyridinyl group and side chain of the three neonicotinoids by the N-dealkylation reaction pathway, resulting in the product in 6-chloro-3-pyridinemethanol and respective side chain fragments. In another study for the remediation of imidacloprid, two bacterial species (*Ochrobactrum thiophenivorans* and *Sphingomonas melonis*) were isolated from cotton cultivated agricultural soil. Several molecular techniques are used for their identification, such as DNA probes, DNA sequencing, protein synthesis, protein and gene expression analysis, nucleic acid extraction, PCR, and DGGE (Erguven and Demirci, 2021).

**FIGURE 8:**
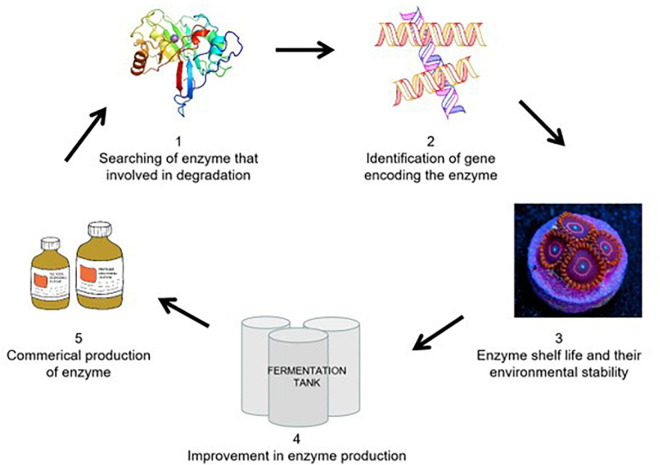
Schematic strategy for the purification of novel enzymes involved in biodegradation.

In order to further elucidate the molecular mechanism, the enzyme-based removal of xenobiotics is a straightforward, quick, eco-friendly, and socially acceptable approach for biodegradation in the natural environment. So, to fulfill this gap, scientists from all over the world have been working continuously to provide the most effective solution to increase environmental pollution (Sharma et al., 2018). A schematic diagram for the purification of novel enzymes by different sources has been illustrated in Figure 8. There are many enzymes that were purified by different sources which are involved in the bioremediation of neonicotinoids enlisted in Table 4.

**TABLE 4:** Enzyme from different sources involved in the biodegradation of neonicotinoid insecticides.

Target neonicotinoid	Enzymes	Source	Comments	References
Imidacloprid, Thiamethoxam and Dinotefuran	CYP6ER1	*Nilaparvata lugens*	Over-expressed in thiamethoxam-resistant and dinotefuran-resistant strains	Pang et al., 2016
Imidacloprid	CYP6G1	*Drosophila melanogaster*	An enzyme that produces toxic but easily excreted metabolites	Fusetto et al., 2017
Imidacloprid	CYP6CM1	*Bemisia tabaci*	*Bemisia tabaci* resistant to imidacloprid lacks resistance to dinotefuran	Hamada et al., 2019
Imidacloprid	CYP353D1v2	*Laodelphax striatellus*	Metabolize imidacloprid to 5 hydroxy-imidacloprid	Elzaki et al., 2017
Thiamethoxam	CYP6CY14	*Aphis gossypii*	RNA interference targeting CYP6CY14 increased the sensitivity of resistant aphid to thiamethoxam	Wu et al., 2018
Acetamiprid and Thiacloprid	Nitrile hydratase	*Ensifer meliloti* CGMCC 7333	Nitrile hydratase transformed the neonicotinoid compounds, and their activity is increased by increasing the concentrations of dichloromethane and hexane	Zhao et al., 2019a
Acetamiprid	CYP5147A3	*Phanerochaete chrysosporium*	The degradation rate of acetamiprid significantly increased and transform into two metabolites	Wang et al., 2019a
Thiacloprid	Nitrile hydratase	*Variovorax boronicumulans* CGMCC 4969	Degrade the thiacloprid and transform into amide metabolite	Zhang et al., 2012
Acetamiprid	Amidase	*Pigmentiphaga sp.*	Amidase hydrolyze the C-N bond of acetamiprid and transform into metabolite IM 1-4	Yang H. et al., 2020
Nitenpyram	Cytochrome P450	*Rhodococcus ruber* CGMCC 17550	The cytochrome P450 mediate the hydroxylation pathway of nitenpyram and 1-aminobenzotriazole strongly inhibited nitenpyram degradation	Dai et al., 2021
Acetamiprid and Imidacloprid	Cytochrome P450 and laccase	*Trametes versicolor*	Cytochrome P450 play a vital role in the transformation of neonicotinoids and proposed degradation pathway	Hu et al., 2021
Thiacloprid	Nitrile hydratase	*Proteobacteria and Acidobacteria*	Biochar altered the soil properties and enhanced the degradation of thiacloprid and activity of nitrile hydratase	Zhang et al., 2018b
Imidacloprid	Dehydrogenase and *ortho-* diphenol oxidase	Commercial	The grass layer in biobed enhances the activity of dehydrogenase and *ortho-* diphenol oxidase which helps the dissipation of pesticides	Delgado-Moreno et al., 2017

For the bioremediation of acetamiprid in surface water, Sun et al. (2017) had used plant growth-promoting rhizobacterium, *Variovorax boronicumulans* CGMCC 4969, and their enzymatic mechanism was also investigated. The bacterium removed 34.7% of 2 mg/L acetamiprid over 120 h with a degradation half-life of 182 h, and the major intermediate was the amide product, (E)-N^2^-carbamoyl-N^1^ -[(6-chloro-3-pridyl) methyl]-N^1^ -methylacetamidine (IM-1-2). Gene cloning and over-expression related studies proved that a nitrile hydratase mediated acetamiprid hydration to IM-1-2. Yang H. et al. (2020) studied the enzymatic degradation pathway of acetamiprid using the *Pigmentiphaga sp.*, facilitated by nitrile hydratase. Additionally, amidase and its encoding genes such as *aceA* and *aceB* are used for effective degradation. In general, *aceB* and a novel amidase showed the ability of initially hydrolyzing the C-N bond of acetamiprid to produce 1-(6-chloropyridin-3-yl)-N-methylmethanamine (IM 1-4), which achieved almost complete degradation of acetamiprid (Figure 3). In another study, Yang W. L. et al. (2020) studied the degradation of sulfoxaflor insecticide by enzymatic mechanism. A bacterium was isolated and identified as *Aminobacter sp.* CGMCC 1.17253 that degraded sulfoxaflor, while recombinant *Escherichia coli* strain protected the *Aminobacter sp.* CGMCC 1.17253 nitrile hydratase gene and the pure nitrile hydratase cobalt-containing with the subunits of α, β, attachment proteins, and three-dimensional homology of nitrile hydratase were obtained. The substrate specificity test of this study further explained that these enzymes play a significant role in other neonicotinoids such as acetamiprid and thiacloprid into their relative amide’s intermediates. By the hydration pathway, the bacterial strains convert sulfoxaflor into their intermediate [N-(methyl(oxido) {1- [6- (trifluoromethyl)pyridin-3-yl] ethyl}-k4-sulfanylidene) urea] (Yang et al., 2021).

Cytochrome P450s played a vital role in the metabolism of organic pollutants using rot fungi (Mori et al., 2017). Wang et al. (2019a) demonstrated the enzyme P450 (enriched by *Phanerochaete chrysosporium*) to degrade neonicotinoid insecticide acetamiprid effectively. After the incubation of 20 days, *P. chrysosporium* eliminate 21% and 51% of acetamiprid in two different media such as ligninolytic and non-ligninolytic, respectively.

Zhou L. Y. et al. (2014) previously reported that the nitrile hydratase enzyme of *Ensifer meliloti* CGMCC 7333 was a powerful tool to convert acetamiprid into the N-amidoamine metabolite, which is unstable and further degrade to make intermediate chlorinated pyridyl methylmethanamine compound. Fascinatingly, *Ensifer meliloti* CGMCC7333 was also proficient at degrading thiacloprid into the N-carbamoyl imine intermediate by using the same enzyme (Ge et al., 2014).

The biodegradation of imidacloprid using *Trichoderma atroviride* strain T23 and screening of transformants to achieve maximum degradation rate was investigated. REMI (restriction enzyme-mediated integration) technique was adopted for the construction of mutant strain. Plasmid pBluescript II KS-*hph* (4334 bp) containing hygromycin (*hph*) and ampicillin (*amp*) resistance genes was used. Finally, REMI mutants were confirmed by PCR and Southern hybridization analysis. Results revealed that total 174 transformants were developed from the wild *T. atroviride* strain T23. During sub-culturing, 21 colonies lost their resistance to hygromycin and the other 153 grew stably with a transformation frequency of 87.9%. The efficiency of transformants for the biodegradation of imidacloprid was investigated by the colorimetric method. Among 153 transformants, 57% of them showed maximum degradation ability compared to wild strain (He et al., 2014). For the bioremediation of acetamiprid in an agroecosystem, Wang et al. (2013a) isolated *Pigmentiphaga sp*. strain AAP-1 from polluted soil. The results demonstrated that bacterial strain degraded acetamiprid in soil efficiently. The bacterial community was also recovered from contaminated soil, by analysis of terminal (RFLP), after the incubation of *Pigmentiphaga sp*. strain AAP-1.

### Omics Approaches

The biodegradation mechanism has certain limitations. It is successful at one location and may not be feasible in other places, and identifying the polluted sites is also quite time-consuming. Additionally, the mechanism that controls the growth and activity of microbial strains to degrade xenobiotics in field conditions is not well understood (Lovley, 2003). Therefore, laborious efforts are needed to make the degradation process effective, faster, quite efficient, and more suitable to act on a wide variety of organic and inorganic pollutants (Kumar Awasthi et al., 2020). By using molecular techniques to protect the environment, including genomics, transcriptomics, proteomics, and metabolomics plays a significant role in microbial habitats (Li et al., 2017).

These omics demonstrate extensive insights into the functional behavior of organisms by enhancing our knowledge of key biosynthetic processes and molecules such as genes, proteins, and metabolites. These approaches not only are used to investigate the crucial role of microbial species that regulate soil functions, enhance plant growth, and are used as a quorum sensing to understand the community composition to assign an ecological role but also provide behavioral information of cultured and uncultured microbial species and identify genes that actively participate in degradation process (Sharma et al., 2008; Festa et al., 2017; Malik et al., 2021). These omics also provide essential data about the genes and proteins involved in the degradation of pesticides and their metabolites (Rodríguez et al., 2020). Biodegradation genomics is used to identify effective genes in various microbial communities that encode specific enzymes used in biodegradation (Bharagava et al., 2019). Recently, Yang H. et al. (2020) identified novel amidase enzymes and genes from *Pigmentiphaga sp.* strain D-2 by using genomics tools involved in the degradation of acetamiprid. In another study, Guo et al. (2021) studied the degradation of acetamiprid by hydration pathway by *Pseudaminobacter salicylatoxidans* CGMCC 1.17248. Furthermore, this study explained that by gene cloning and overexpression, the identification of two different nitrile hydratase AnhA and AnhB simultaneously converted acetamiprid into {1-[(6-chloro-pyridin-3-ylmethyl)-methyl-amino]-ethylidene}-urea (IM 1-2). Proteomic analysis of microbial species provides an understanding of the protein pattern, function, interaction, and regulation (Jarnuczak et al., 2019).

The identification and physiological state of microbial communities can assist the understanding of genes that are associated with biodegradation mechanism and their regulation process (Kohl, 2020). Sun et al. (2018) identified two different NHase genes (*cnhA* and *pnhA*) by *Ensifer adhaerens* CGMCC 6315 to degrade acetamiprid, while proteomic analysis showed that the upregulation expression of *pnhA* genes improved degradation of acetamiprid ability. Transcriptomics is a prevailing tool used to assess microbial RNA expression and regulation on a whole organism level and provide an extensive collection of small regulatory non-coding RNAs (Hör et al., 2018). Recently, Wang et al. (2021) provided a novel study for assessing of neonicotinoids on neuro-2a cells by lipidomics and metabolomics, which provides the ecological risk of neonicotinoids and contributes to investigating their residues in animals and humans in the future.

However, unraveling microbial interactions in complex microbial communities is a challenging task. Adopting these multi-omic approaches in co-existence with culture-based confirmation technique efficiently explains the microbial interactions affecting the biodegradation processes. It contributed to the future application and operation of environmental bioprocesses on a knowledge-based control (Chandran et al., 2020).

### Database Approaches

Nowadays, bioinformatics and computational biology have been receiving wide attention in scientific research for the solution of biological problems. These techniques used biological principles with the fusion of mathematical, computer, and statistical principles to assist the development and application of bioremediation (Tomar, 2021). Besides the molecular and omics approaches, many online databases provide information regarding pesticide biodegradation by using microbes and their pathways (Nolte et al., 2018). The mainly used databases in the field of biodegradation are the University of Minnesota pathway prediction system (UM-PPS), microbial volatile organic compounds (mVOCs), University of Minnesota biocatalysts or biodegradation database (UM-BBD), biodegradation network molecular biology database (BioNeMo), pesticide target interaction database (PTID), microbial genome database (MBGD), biodegradative oxygenase’s database (oxdbase), and biocyc and metacyc compatible with both Windows and Linux operating system (Hou et al., 2004; Effmert et al., 2012; Arora and Bae, 2014). UMBBD pathway forecast database shows the data regarding microbial biocatalytic reactions, biodegradation pathways, and metabolites achieved during the removal of pesticides by microbes (Dvořák et al., 2017).

University of Minnesota pathway prediction system provides information about the possible intermediates of pesticides, biocides, and pharmaceuticals. Various researchers such as environmental microbiologists, risk assessors, and analytical chemists used this database and discovered most related metabolites (Wicker et al., 2010). The mVOCs database provides information about compound identification and allows quick mass spectrum comparison. Moreover, it offers new insights into the generation of microbial volatiles, which helps in various fields such as quorum sensing and medical applications (Lemfack et al., 2018). The microbial genome database plays a significant role in the inspection at the genomic level used for estimating the positions of genes, ortholog recognition, and collection of paralog data (Parks et al., 2015). The biodegradative oxygenase’s database was developed by the CSIR Institute of Microbial Technology, Chandigarh, India. This database stores information regarding oxygenase enzymes that take part in the degradation process from published literature and databases (Arora et al., 2009). Biodegradation network molecular biology databases have access to sequences barcoding for biodegradation genes and their transcription and regulation (Kumari and Kumar, 2021). For the biodegradation of thiamethoxam, a database approach was studied by Wu et al. (2021), and the biodegradation protein database including 17 sub-databases (*alkb*, *benA*, *bph*, *bphA*1, *bphA*2, *carA*, *dbfA*1, *dxnA*, *dxnA-dbfA*1, *glx*, *lip*, *mmox*, *mnp*, *npah*, *ppah*, *ppo*, and genes of P450 enzyme) was downloaded from the FunGene protein database^2^. Besides this, the pesticide degradation gene protein database was also recovered from the NCBI protein database for eight neonicotinoid pesticides, including acetamiprid, imidacloprid, thiamethoxam, clothianidin, dinotefuran, flonicamid, clothianidin, and nitenpyram.

### Immobilization Techniques

Immobilization is considered the most efficient technique for utilizing microbial isolates in continuous mode (Ugwuodo and Nwagu, 2020). Immobilization stops the misplacement of microbial strains during uninterrupted operation and enhances the cell density, which plays a significant role in the degradation process. Moreover, the immobilized microbes can withstand extreme environmental stress such as temperature, pH, and toxic compounds (Kurade et al., 2019). For immobilization, the support substances are vital as well as biocompatible with microbial cells and their enzymes. Different types of substances like organic (alginate, chitosan, agar, polyvinyl alcohol, collagen, cellulose, keratins, carrageenan, and chitin), inorganic (silica, alumina, iron oxides, and carbon-based materials), organic-organic hybrid, and organic-inorganic hybrid substances have been used to immobilize bacteria and other microbes (Horchani et al., 2012; Zdarta et al., 2019). Besides these substances, clay materials (bentonite, halloysite, kaolinite, montmorillonite, and sepiolite) and new support materials such as magnetic particles, mesoporous materials (zeolites, carbons, and sol-gel matrices), nanoparticles (nanogold, silver, iron, and graphene), ceramic materials (alumina, zirconia, titania, silica, iron oxide, and calcium phosphate), and electrospun materials are reported in for the immobilization of microbial cells and enzymes (Zdarta et al., 2018).

Among them, inorganic substances that have adequate mechanical strength in aggregation with synthetic or natural organic materials are more suitable for excessive biocompatibility (Wang Y. et al., 2020). There are different geometric shapes of immobilized materials such as beads (Liu et al., 2019), hollow cylinders (Lee et al., 2016), and core-shell structure beads that have been reported (Yu et al., 2019).

**FIGURE 9:**
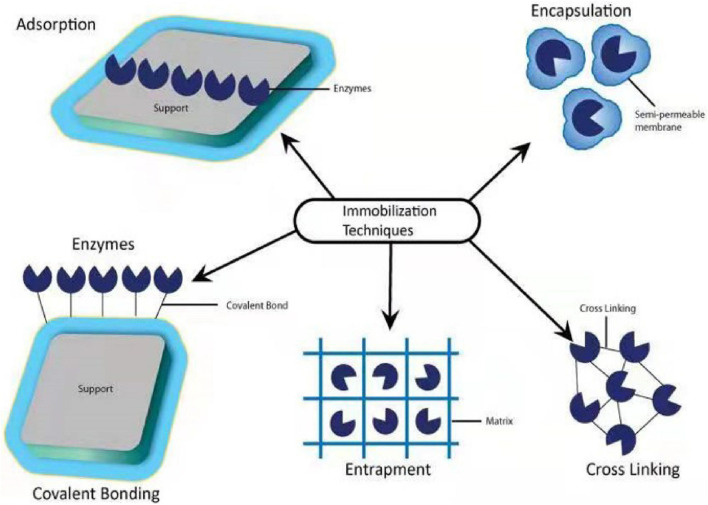
Immobilization methods for neonicotinoid-degrading microbes and enzymes.

The immobilization technique can be done with the help of physical and chemical methods such as physical adsorption, entrapment, encapsulation, covalent bonding, and cross-linking (Wang W. et al., 2020; Figure 9). In physical adsorption, the microbial cells are attaching to the surface of the support by the poor van der Waals and *via* different interactions such as electrostatic, hydrophobic, and hydration, in which cells are vulnerable to the culture solution immediately (Sandhyarani, 2019).

For the process of entrapment, microbial cells are encapsulated within the polymeric matrix. Encapsulation maintained microbial strength with a semipermeable membrane capable of supplying nutrients and substrates between the carrier substances and culture media (Kumar et al., 2016). In all these methods, entrapment and encapsulation using organic or inorganic polymers is a ubiquitous method for separating bacterial cells from the growing environment (Jia et al., 2014). To remove imidacloprid residues in water, Ma et al. (2021a) synthesized a novel and efficient magnetic sludge biochar (CoFe_2_O_4_) with the modification of graphene oxide. Kinetics, isotherms, thermodynamics, and environmental factors analysis demonstrated that both physisorption and chemisorption were involved in the imidacloprid adsorption onto graphene oxide metal sludge biochar. The adsorption capacity of CoFe_2_O_4_ ensured the magnetic sensitivity of graphene oxide magnetic sludge biochar, which enabled it to be easily separated from water solution and could be used to remove the residues of imidacloprid from polluted environment. In another study, Ma et al. (2021b) adopted another way to degrade imidacloprid in water by choosing sugarcane bagasse due to its higher adsorption capacity of 313 mg/g at 298 K synthesized in potassium hydroxide and other magnetic particles (iron/zinc, Fe/Zn). There are several factors such as characterization, kinetic, isotherm, thermodynamic, and environmental factors analyzed. These factors indicated that both chemisorption and physisorption were spontaneous, endothermic, and randomly increasing processes involved in imidacloprid adsorption. Furthermore, this study demonstrated that magnetic particles easily separated from the solution, which could be reused. This study suggested that magnetic particle-based sugarcane bagasse biochar is an effective, green, and sustainable adsorbent for neonicotinoid biodegradation.

To investigate acetamiprid and silica nanoparticle transportation in pure and biochar amended sands, Wang H. et al. (2016) conducted an experiment. The results demonstrated that the retention of acetamiprid at neutral pH and less ionic strength was less in the pure sand compared to biochar amended sand. Moreover, due to their nonionic attributes, the acetamiprid cannot bond with the biochar by protonation or deprotonation, and the resulting sorption rate was not affected by environmental conditions. In another study, Gomaa et al. (2020) had immobilized *Lysinobacillus macrolides* strain MSR-H10 with sodium alginate for the biodegradation of acetamiprid in clay soil. The results reported that bacterial cells immobilized with sodium alginate degrade rapidly and effectively without lacking their efficiency. Recently, Dai et al. (2021) had isolated actinomycetes *Rhodococcus ruber* CGMCC 17550 from a nitenpyram production sewage treatment tank for surface water treatment. The cells of *Rhodococcus ruber* CGMCC 17550 were immobilized in calcium-alginate, which degraded nitenpyram up to 87.11% with the concentration of 100 mg/L in 8 days. By resulting the biodegradation of nitenpyram, it converts into their three metabolites by a novel hydroxylation pathway (1) (E)-1-((1-(methylamino)-2-nitrovinyl) (pyridin-3-ylmethyl) amino) ethan-1-ol, (E)-6-chloro-3-((ethyl(1-(methylamino)-2-nitrovinyl) amino) methyl) pyridin-2-ol, and (E)-(6-chloropyridin-3-yl) (ethyl(1-(methylamino)-2 nitrovinyl) amino) methanol. Further oxidation cleavage of (E)-(6-chloropyridin-3-yl) (ethyl(1-(methylamino)-2 nitrovinyl) amino) methanol converts into (*E*)-*N*-ethyl-*N′*-methyl-2-nitroethene-1,1-diamine (Figure 5).

Covalent binding and crosslinking are chemical methods for microbial immobilization based on covalent bonds between the functional groups present in the microbial cell wall and its support. Immobilization by the cross-linking process applies multifunctional reagents to encourage the development of a channel between functional groups on the external cell membrane (Giese et al., 2020). In covalent binding and cross-linking methods, whole microbial cells are vulnerable to chemicals and acute conditions, which may injure the cell surface and decline its metabolic activity. Therefore, crosslinking has been more victorious in the immobilization of non-viable microbial cells (Martins et al., 2013). Cross-linking has a wide range of acceptance for microbial immobilization and presents benefits like speed and simplicity compared to covalent bonding, and is challenging to control carefully (Mehrotra et al., 2021). By using covalent bonding and adsorption, Chen et al. (2019) had immobilized laccase enzyme with the support of wheat straw and peanut shells to remove nitenpyram in the agroecosystem. The results revealed that the successful application of immobilized laccase enzymes for the degradation of nitenpyram in agroecosystem has a strong and effective potential.

The half-life of the immobilized enzyme was reported to be higher than the soluble enzyme, with only a slight decrease in the catalytic activity of up to 12 consecutive cycles (Bhatt et al., 2020). The enzyme immobilization technique aims to enhance the stability of enzymes that depend on the structure, method of immobilization, and matrix material. Enzymes purified from diverse microbes can be a superior choice for large-scale pesticide biodegradation in a short time. The choice of immobilization technique in biodegradation plays a significant role with cost-effective, stable performance, good mass transfer, high intensity, long lifespan, and environment friendly (Hameed and Ismail, 2020; Celik et al., 2021).

## Conclusion and Future Perspectives

Due to the imprudent use of neonicotinoids throughout the world, their residues increase in the environment, causing severe threats for non-target organisms like bees. The biodegradation of neonicotinoid pesticides in the environment through microbes associated with their enzymes and genes in the field of molecular basis has come forth as an excellent option. There are many bioremediation techniques and strategies available to alleviate neonicotinoid risks. In the microbial degradation process, the elements including catabolic genes, enzymes, plasmids, proteins, mobile elements, and transposons play essential roles to catalyze novel biochemical pathways. Furthermore, omic approaches have the potential to forecast microbial communities and their metabolism in contaminated sites. These omic approaches also pay many benefactions to the logical identification of the potential microbes. These microbial technologies track novel microbes, providing new and excellent insights into their critical bio-degrative pathways at the molecular level. The immobilization of microbes with various carrier materials makes them more tolerant against the toxicity of hazardous pollutants. It provides a large specific area, strong adsorption ability, high porosity, and permeability of microorganisms. Recent advances in microbial technologies degradation of toxic compounds prediction systems have made tremendous and efficient advancements that allow virtual screening and toxicity profiles of hazardous pollutants. Except for these approaches, more attention is required to the effective use of genetic tools such as genetic libraries, genetic fingerprinting, radio respirometry, and micro autoradiography in neonicotinoid polluted environments. Besides, the progress of rapid genomic tools, bioinformatics tools, artificial intelligence, and system biology can be additionally used to inspect covered or invisible microbes in a polluted environment. Moreover, a large number of genetically modified organisms (GMOs) are required to construct because indigenous microbes are not active in every environment and GMOs play a crucial role in the biodegradation of xenobiotics. Various innovative techniques could be applied to produce GMOs, such as molecular cloning, horizontal transfer of DNA, electroporation and protoplast transformation, biolistic transformation, conjugation, and transformation of most capable microbial cells. To degrade different pesticide groups on an industrial scale, biochar production, enzymes production using fermentation chamber, and cost-effective carrier materials used in bioreactor systems are seeking wide attention.

## Author Contributions

JL and GZ conceived the presented idea. SA prepared the original manuscript, figures, and tables. SA, JL, GZ, and DC contributed to revising the manuscript. JL supervised the project. All authors approved it for publication.

## Conflict of Interest

The authors declare that the research was conducted in the absence of any commercial or financial relationships that could be construed as a potential conflict of interest.

## Publisher’s Note

All claims expressed in this article are solely those of the authors and do not necessarily represent those of their affiliated organizations, or those of the publisher, the editors and the reviewers. Any product that may be evaluated in this article, or claim that may be made by its manufacturer, is not guaranteed or endorsed by the publisher.

## Footnotes

1 https://sitem.herts.ac.uk/aeru/ppdb/en/index.htm, accessed: 23 May 2021.

2
http://fungene.cme.msu.edu
